# Supplementary material for: AAV2/9-mediated silencing of PMP22 prevents the development of pathological features in a rat model of Charcot-Marie-Tooth disease 1 A
Source: Nat Commun. 2021 Apr 21;12:2356. doi: 10.1038/s41467-021-22593-3 (PMC8060274; doi:10.1038/s41467-021-22593-3)
Supplement: Supplementary file 1 — Supplementary Information [file 41467_2021_22593_MOESM1_ESM.pdf]

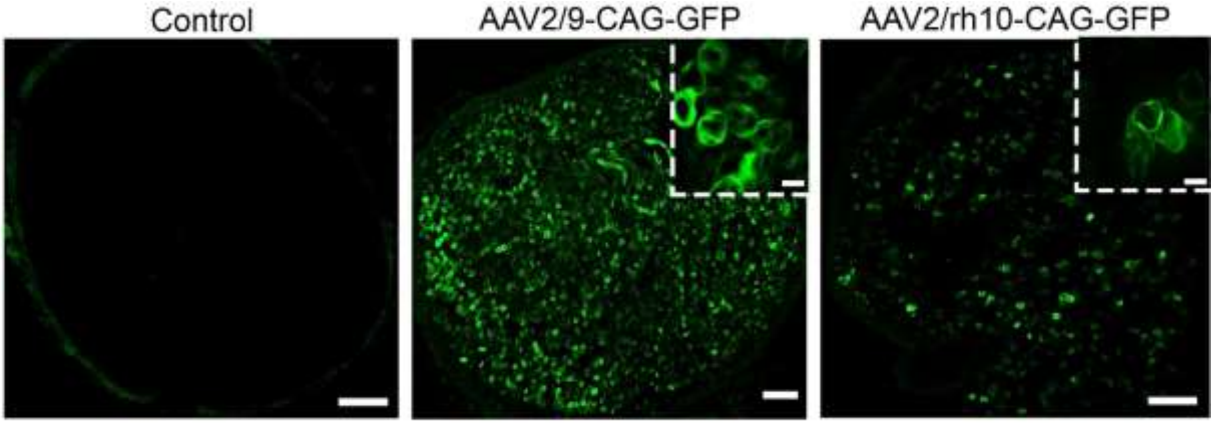


**Fig. S1 AAV2/9 and AAV2/rh10-CAG-GFP transduce mSC after intra-nerve injection in mouse**. Representative images of sciatic nerve cross-sections showing GFP protein expression at the injection site after intra-nerve injection of AAV2/9 and AAV2/rh10-CAG-GFP in adult mouse (2-3 months old, 5 x $10^{10}$ vg/nerve in 8 µl, *n* = 3 animals per group). Control represents an adult mouse sciatic nerve injected with Fast Green alone. All animals were sacrificed one month post-injection. Inserts show the circular shape of transduced cells. Scale bars: 100 µm and 10 µm for the inserts.

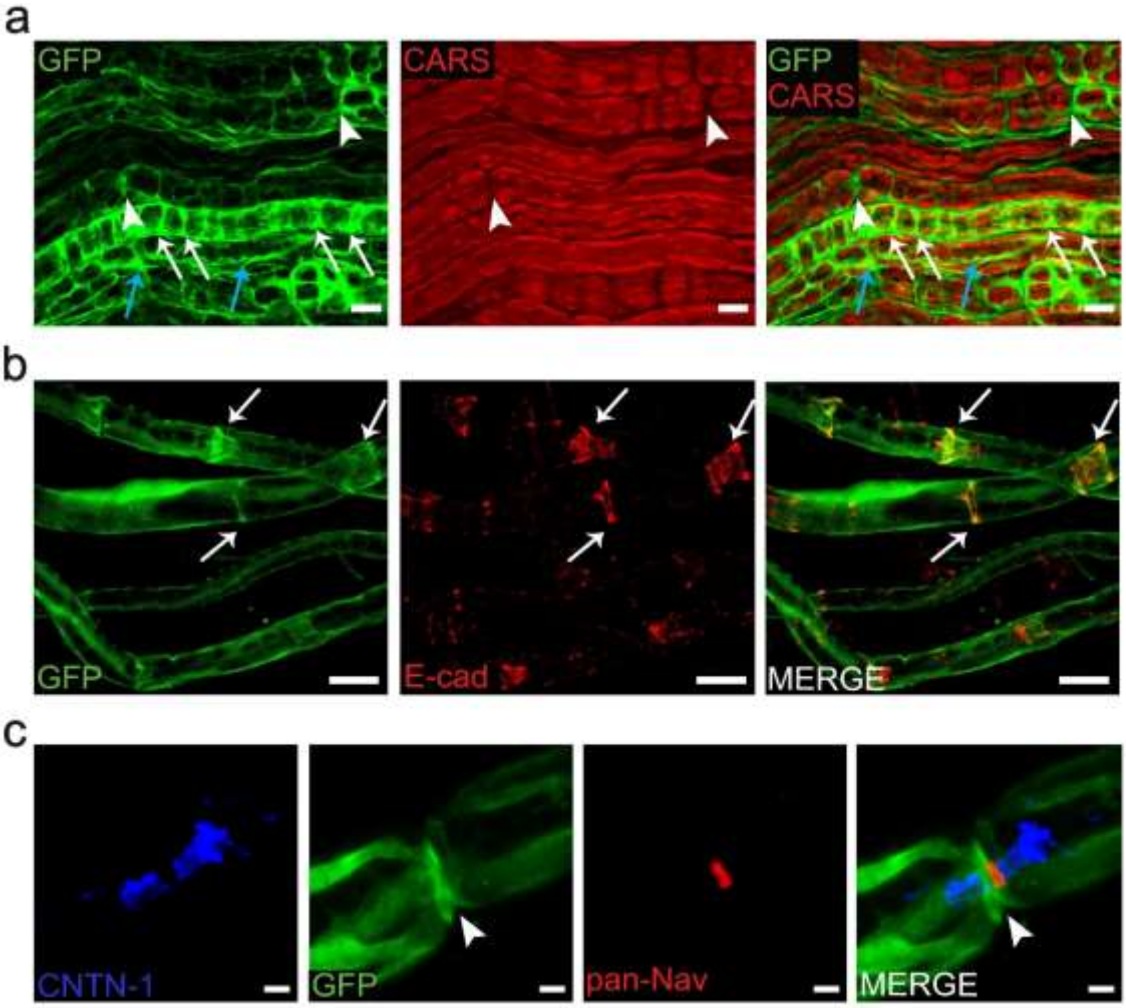


**Fig. S2 GFP is expressed in mSC after intra-nerve injection**. **a** GFP (green) and CARS (red) imaging of sciatic nerve injected with AAV2/9 expressing GFP in rat pup (1 x $10^{11}$ vg/nerve in 8 µl, *n* = 3 animals) twelve months post-injection showing specific features of transduced mSC, such as Schmidt-Lanterman incisures (white arrows), Cajal's band (blue arrows) and paranodal loops surrounding nodes of Ranvier (arrrowheads). Scale bar: 10 µm. **b** and **c** Representative images of immunostainings on teased fibers from a rat sciatic nerve injected with AAV2/9-CAG-GFP (injection at P6-P7, 1 x $10^{11}$ vg/nerve in 8 µl, *n* = 3 animals) and sacrificed one month post-injection. GFP protein is expressed in Schmidt-Lanterman incisures immunostained with E-cadherin (E-cad, red) (**b**) and paranodal loops surrounding nodes of Ranvier immunostained with pan-Nav (red) and paranodes immunostained with Contactin-1 (CNTN-1, blue) (**c**). Scale bars: 10 µm (**b**) and 2 µm (**c**).

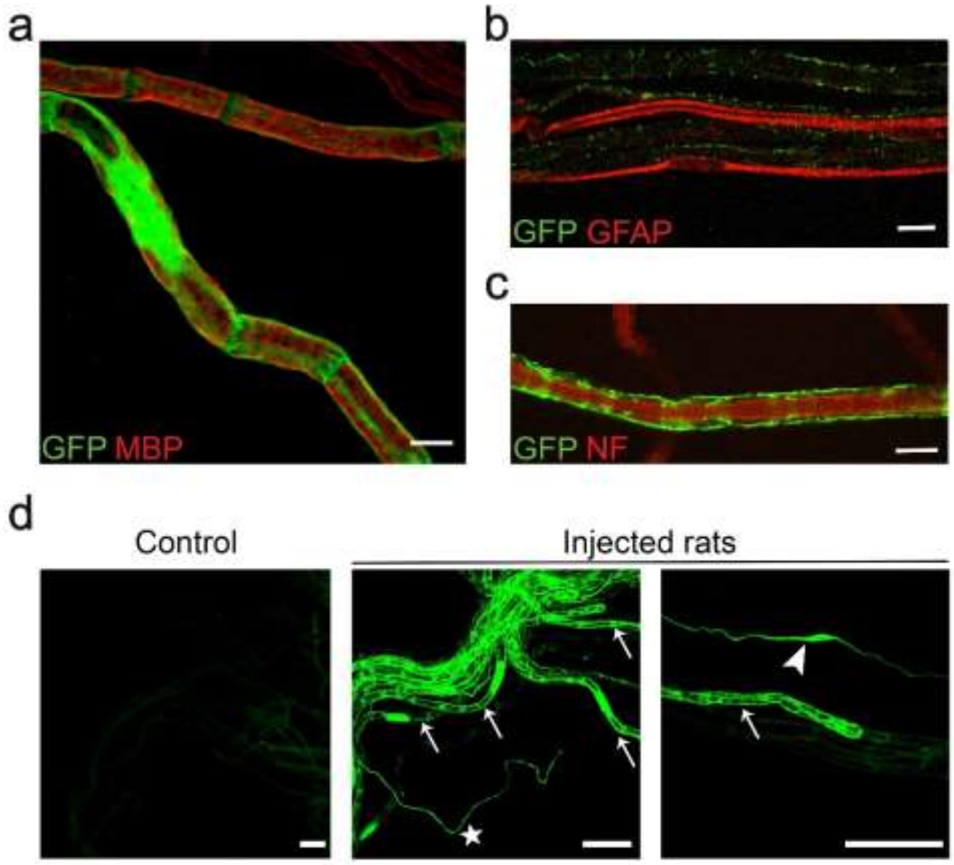


**Fig. S3 AAV2/9-CAG-GFP transduces more specifically mSC than other cells after intra-nerve injection**. Representative images of teased fibers from a rat sciatic nerve injected with AAV2/9-CAG-GFP (injection at P6-P7, 1 x $10^{11}$ vg/nerve in 8 µl, *n* = 3 animals) and sacrificed one month post-injection. Immunostainings with MBP (**a**), GFAP (**b**) and Neurofilament (NF) (**c**) as markers for mSC, nmc and axon respectively show that GFP labels mSC. Scale bars: 10 µm. **d** In addition to mSC (arrows), GFP labels some nmc (arrowhead) and axons (stars). Control represents a rat pup sciatic nerve injected with Fast Green alone. Scale bars: 50 µm.

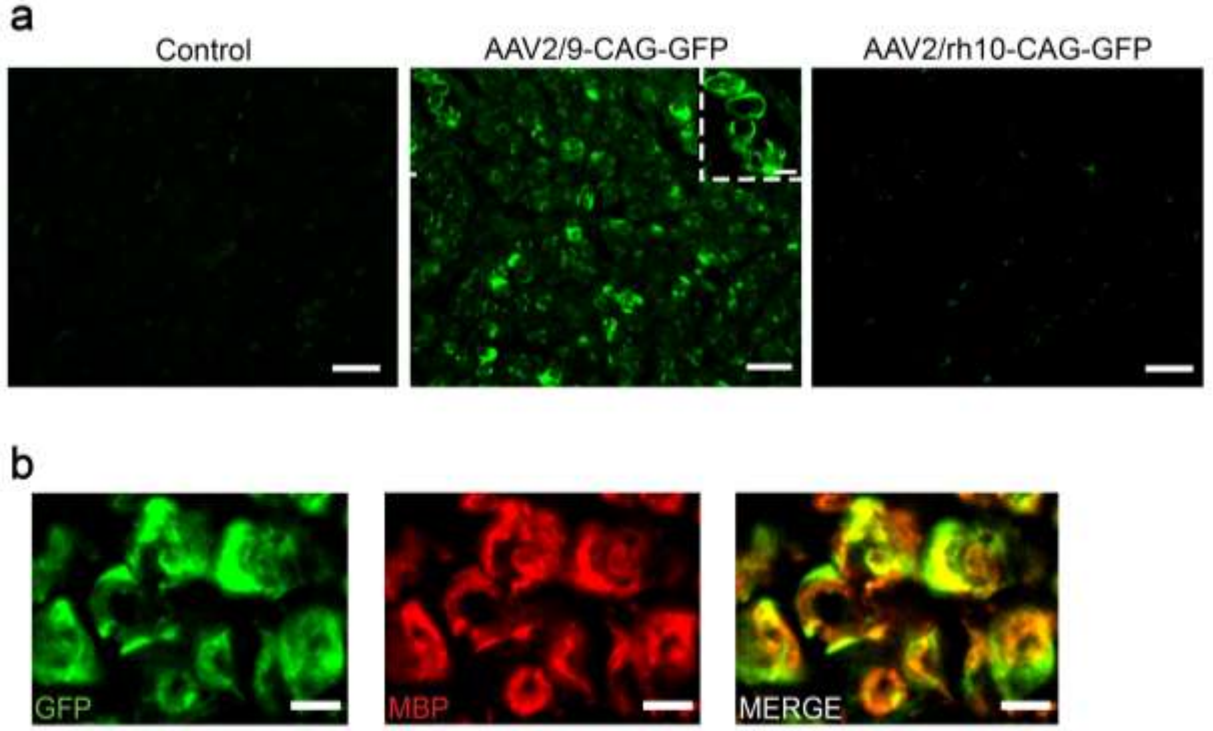


**Fig. S4 AAV2/9-CAG-GFP transduces mSC more efficiently than AAV2/rh10-CAG-GFP after intra-nerve injection in NHP sciatic nerves. a** Representative images of sciatic nerve cross-sections showing GFP protein expression at the injection site after intra-nerve injection of AAV2/9 and AAV2/rh10-CAG-GFP in adult NHP (3.7 and 2.3 years old for AAV2/9 and AAV2/rh10, 5 x $10^{12}$ vg/nerve in 416 µl, *n* = 1 animal per group). Control shows the contralateral sciatic nerve. All animals were sacrificed one month post-injection. Inserts show the circular shape of transduced mSC. Scale bars: 50 µm and 10 µm for the inserts. **b** Representative images of immunostaining on sciatic nerve cross-sections of adult NHP at the injection site using myelin MBP marker (red) (*n* = 3 nerve cross-sections per NHP). GFP (green) partially colocalizes with MBP. Scale bar: 10 µm.

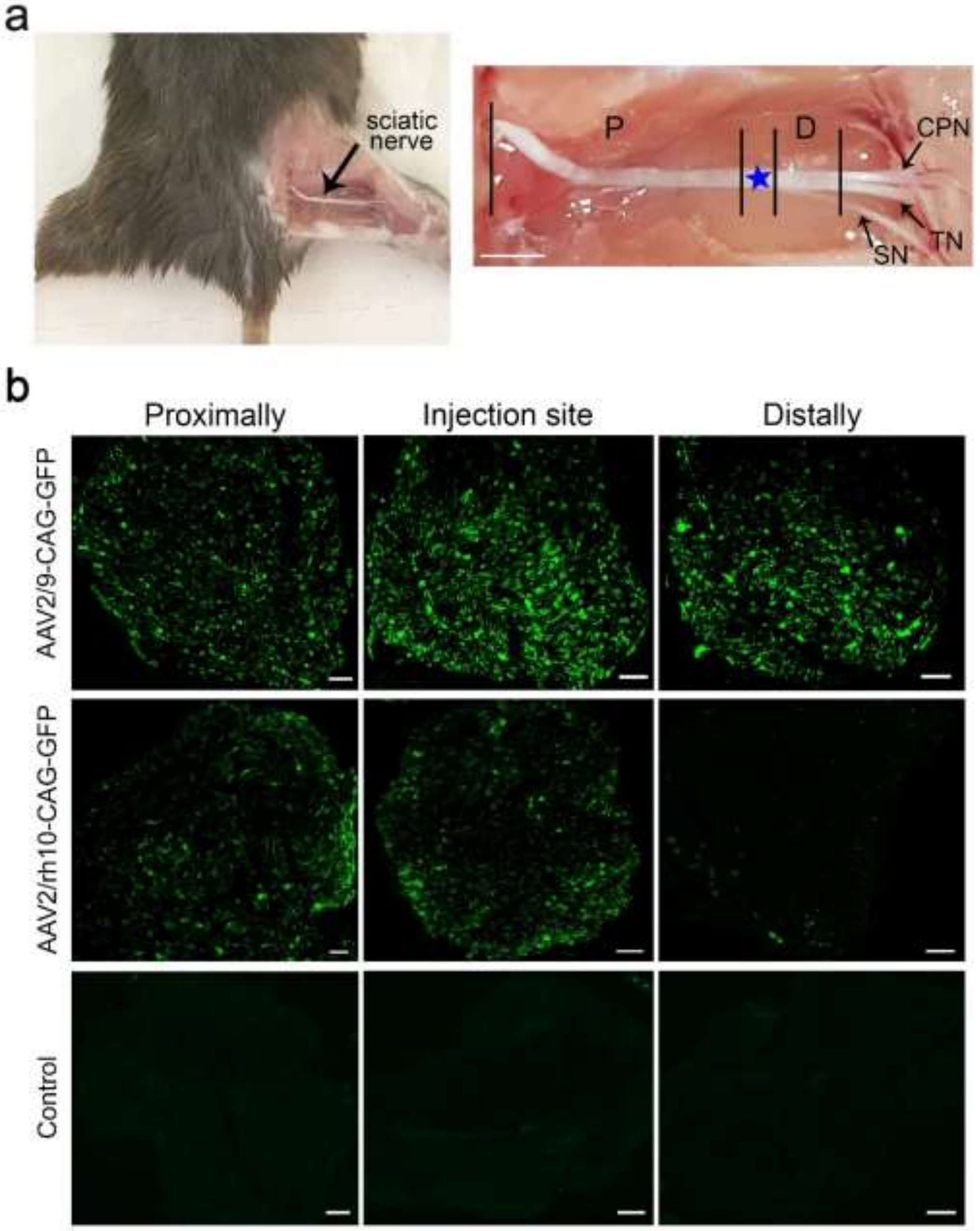


**Fig. S5 AAV2/9-CAG-GFP exhibits a higher diffusion along the sciatic nerve than AAV2/rh10-CAG-GFP mSC after intra-nerve injection in rodents. a Left:** Dorsal view of a mouse showing the localization of the right sciatic nerve. **Right**: Higher magnification of the right sciatic nerve showing the injection site (blue star) and the proximal (P) and the distal (D) parts. CPN: Common peroneal nerve, TN: tibial nerve, SN: sural nerve. Scale bar: 0.5mm. **b** Representative images of sciatic nerve cross-sections showing GFP protein expression at the injection site, proximally and distally, after intra-nerve injection of AAV2/9 and AAV2/rh10-CAG-GFP in rat pup (P6-P7, 1 x $10^{11}$ vg/nerve in 8 µl, *n* = 3 animals per group). Control represents a rat pup sciatic nerve injected with Fast Green alone. All animals were sacrificed one month post-injection. Scale bars: 50 µm.

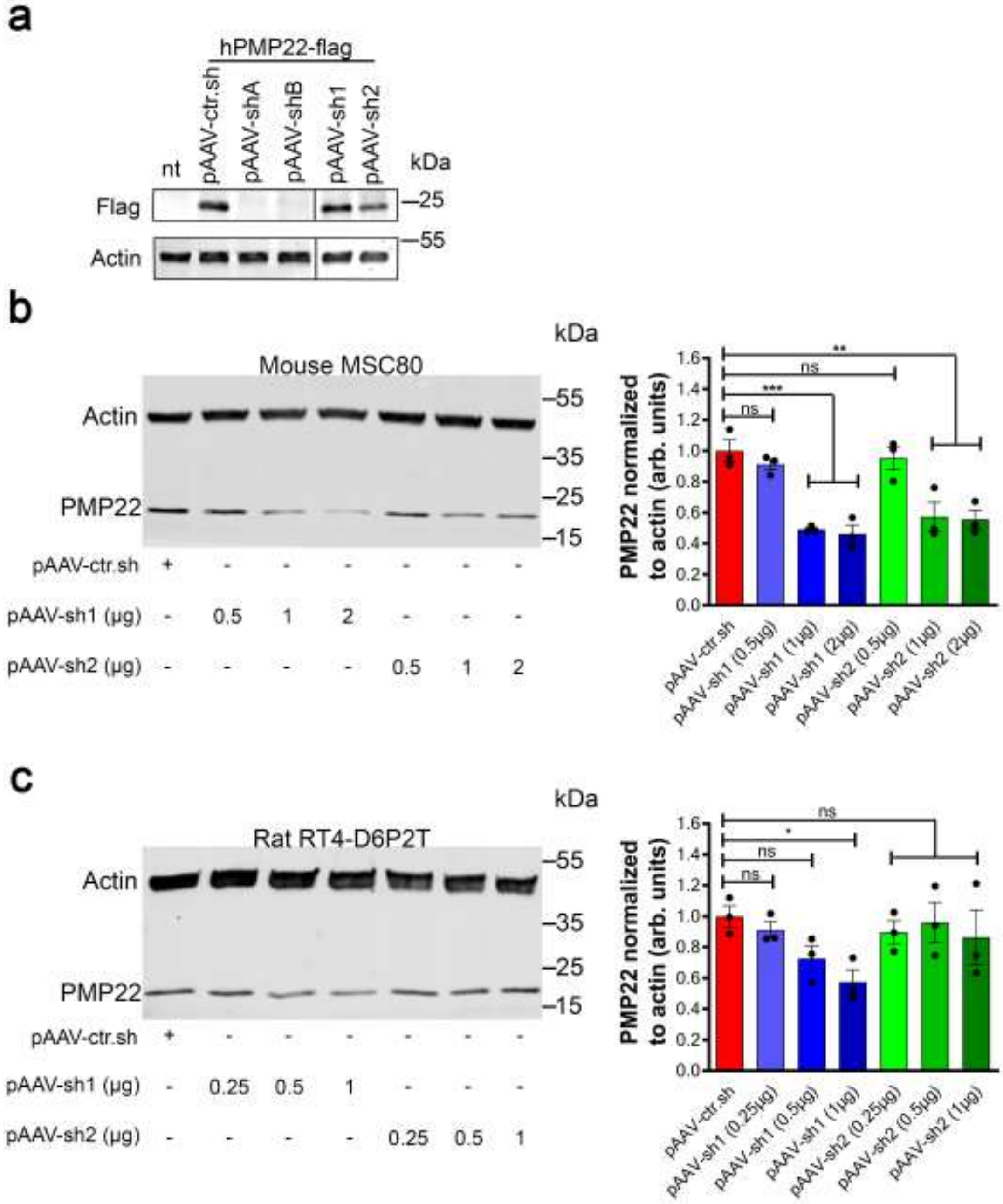


**Fig. S6 *In vitro* validation of shRNAs targeting human or rodent PMP22 expression. a** Representative Western blot of three independent experiments showing human Flag-tagged PMP22 detected with Flag antibody (Flag) and β-actin as loading control (Actin). HEK293 cells were non-transfected (nt) or cotransfected with pCMV3 human Flag-tagged PMP22 plasmid (hPMP2-Flag) and pAAV-ctr.sh, pAAV-shA, pAAV-shB (both targeting human *PMP22* mRNA), pAAV-sh1 or pAAV-sh2 (both targeting mouse *Pmp22* mRNA). All bands were obtained on the same membrane but not contiguously (black line separates the two parts). **b Left**: Western blot analysis showing mouse PMP22 protein expression in mouse MSC80 Schwann cell line transfected with pAAV-sh1, pAAV-sh2 or pAAV-ctr.sh at the indicated dose per well (µg of DNA). Actin expression is used as loading control. **Right**: quantitation of mouse PMP22 expression normalized to actin (*n* = 3 independent experiments). Statistical tests

show one-way ANOVA followed by Dunnett's post hoc test, two-sided. *** $p = 0.0003$ between pAAV-ctr.sh and pAAV-sh1 (1 µg), *** $p = 0.0002$ between pAAV-ctr.sh and pAAV-sh1 (2 µg), ** $p = 0.0014$ between pAAV-ctr.sh and pAAV-sh2 (1 µg) and *** $p = 0.001$ between pAAV-ctr.sh and pAAV-sh2 (2 µg); ns, not significant; arb. units, arbitrary unit. All error bars show SEM **c Left**: Western blot analysis of rat PMP22 protein expression in rat RT4-D6P2T Schwann cell line transfected with pAAV-sh1, pAAV-sh2 or pAAV-ctr.sh at the indicated dose per well (µg of DNA). Actin expression is used as loading control**. Right**: quantitation of mouse PMP22 expression normalized to actin ($n = 3$ experiments independent). Statistical tests show one-way ANOVA followed by Dunnett's post hoc test, two-sided. * $p = 0.0495$ between pAAV-ctr.sh and pAAV-sh1 (1 µg); ns, not significant; arb. units, arbitrary unit. All error bars show SEM. Source data are provided as a Source Data file.

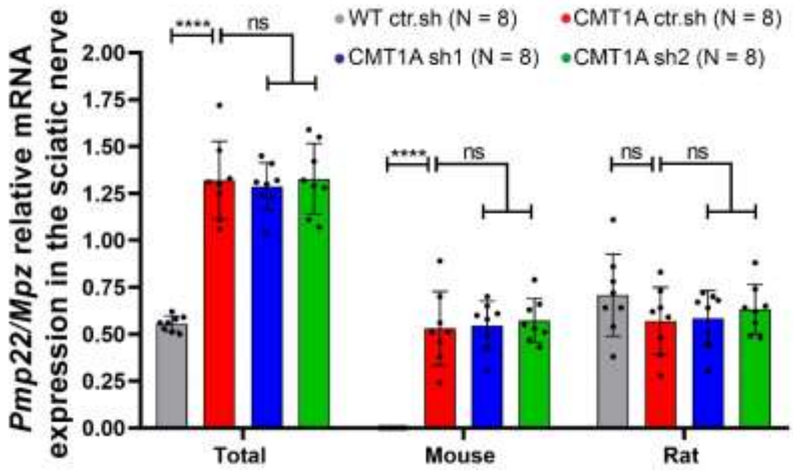


**Fig. S7 Intra-nerve treatments with AAV2/9-sh1 and -sh2 do not affect *Pmp22* mRNA expression in rat CMT1A sciatic nerves.** Mean level of total *Pmp22,* mouse *Pmp22* and rat *Pmp22* mRNA normalized on rat *Mpz* mRNA level in sciatic nerve of WT ctr.sh, CMT1A ctr.sh, CMT1A sh1 or CMT1A sh2 three months after injection. ($n$ = 8 animals per group). *ActB* and *Rsp9* mRNA levels were used as reference genes. Statistical test shows one-way ANOVA followed by Dunnett's post hoc test, two-sided. For total *Pmp22*/*Mpz* relative mRNA expression, **** $p < 0.0001$ between WT ctr.sh and CMT1 ctr.sh; for mouse *Pmp22*/*Mpz* relative mRNA expression, **** $p < 0.0001$ between WT ctr.sh and CMT1 ctr.sh; ns, not significant. All error bars show SD. Source data are provided as a Source Data file.

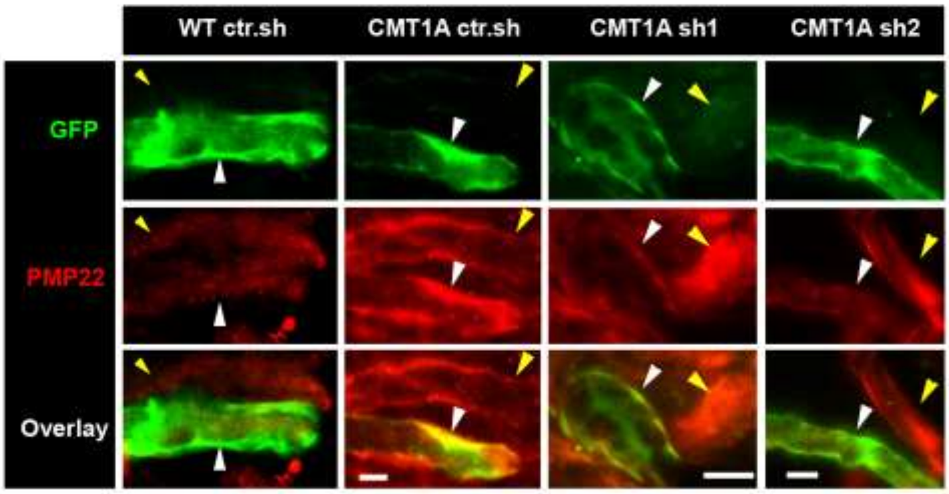


**Fig. S8 Intra-nerve injections of AAV2/9-sh1 and sh2 decrease PMP22 expression in CMT1A rat mSC**

Representative images (*n* = 3 animals per group) obtained from cryo-sections of WT ctr.sh, CMT1A ctr.sh, CMT1A sh1 and CMT1A sh2 sciatic nerves were stained for PMP22 (red) and imaged using a confocal microscope. White arrowheads show transduced mSC expressing GFP (green) and yellow arrowheads non-transduced mSC. In WT ctr.sh and CMT1A ctr.sh nerves transduced cells show a similar amount of PMP22 as non-transduced cells. In CMT1A sh1 and CMT1A sh2 nerves transduced cells show less PMP22 than non-transduced cells, showing that AAV2/9-sh1 and -sh2 decrease PMP22 expression in CMT1A rat mSC. Animals were sacrificed three months post-injection. Scale bars= 5 µm.

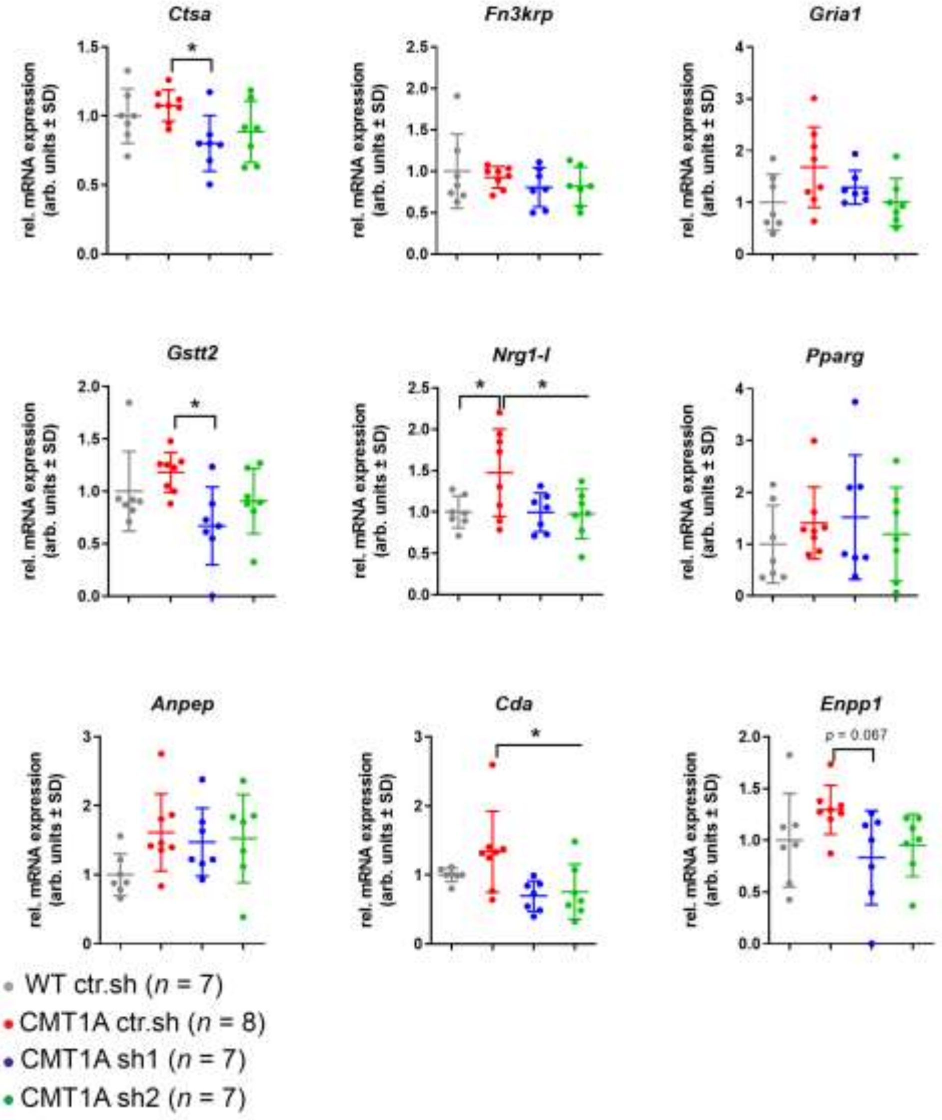


**Fig. S9 Transcriptomic analysis of nine biomarkers in the front paw skin.** The mRNA expressions of nine biomarkers (label on the top of each graph) were quantified using RT-qPCR in the skin of front paws ($n = 7$ animals for WT ctr.sh, $n = 8$ animals for CMT1A ctr.sh, $n = 7$ animals for CMT1A and $n = 7$ animals for CMT1A sh2) and normalized against the mRNA expression levels of the stable housekeeping gene *ActB*. Relative (rel.) mRNA expressions are are expressed as the mean in arbitrary unit (arb. units). Error bars show SD. Statistical tests show one-way ANOVA followed by Sidack's post hoc test, two-sided. For *Ctsa*, * $p = 0.0262$ between CMT1A ctr.sh and CMT1A sh1; for *Gstt2*, * $p = 0.0139$ between CMT1A ctr.sh and CMT1A sh1; for *Nrg1-I*, * $p = 0.0429$ between WT ctr.sh and CMT1A ctr.sh , * $p = 0.0409$ between CMT1A ctr.sh and CMT1A sh1, * $p = 0.0324$ between CMT1A ctr.sh and CMT1A sh2; for *Cda*, * $p = 0.0107$ between CMT1A ctr.sh and CMT1A sh1, * $p = 0.0226$ between CMT1A ctr.sh and CMT1A sh2. When statistical test results are not shown, this indicates non-significant results. Source data are provided as a Source Data file.

**Supplementary Table S1**

| | | *Transduction rate in adult NHP (%)* | |
|---|---|---|---|
| | | AAV2/9-CAG-GFP | AAV2/rh10-CAG-GFP |
| **Proximally** | 4 cm from injection site | 21 ± 2 | < 1 |
| | 2 cm from injection site | 55 ± 4 | < 1 |
| **Injection site** | | 68 ± 3 | < 1 |
| **Distally** | 2 cm from injection site | 69 ± 5 | < 1 |

**Quantification of the transduction pattern after intra-nerve injections of AAV2/9 and AAV2/rh10-CAG-GFP in NHP**. Adult NHP were injected at 3,7 and 2.3 years old with AAV2/9 and AAV2/rh10-CAG-GFP respectively. All animals were sacrificed one month post-injection. The transduction rate is the percentage of transduced mSC (GFP and MBP positive cells) on the overall number of mSC (MBP positive cells) per section. Proximally distances of the injection were 2 and 4 cm. Distal distance of the injection site was 2 cm. The results are expressed as the mean ± SD. Source data are provided as a Source Data file.

**Supplementary Table S2 Multiple comparison tests for data presented in Fig. 5**

**NCV (m/s)**

| | |
|---|---|
| Within each row, compare columns (simple effects within rows) | |
| Number of families | 6 |
| Number of comparisons per family | 6 |
| Alpha | 0,05 |

| Tukey's multiple comparisons test, two-sided | Mean Diff, | 95,00% CI of diff, | Significant? | Summary | Adjusted *p*-Value |
|---|---|---|---|---|---|
| 1 month | | | | | |
| WT scr.-shRNA vs. CMT1A scr.-shRNA | 23,23 | 19,58 to 26,88 | Yes | **** | <0,0001 |
| WT scr.-shRNA vs. CMT1A shRNA49 | 3,244 | -1,639 to 8,128 | No | ns | 0,2451 |
| WT scr.-shRNA vs. CMT1A shRNA50 | 4,830 | -0,7567 to 10,42 | No | ns | 0,0959 |
| CMT1A scr.-shRNA vs. CMT1A shRNA49 | -19,99 | -24,68 to -15,29 | Yes | **** | <0,0001 |
| CMT1A scr.-shRNA vs. CMT1A shRNA50 | -18,40 | -23,85 to -12,94 | Yes | **** | <0,0001 |
| CMT1A shRNA49 vs. CMT1A shRNA50 | 1,586 | -4,492 to 7,663 | No | ns | 0,8630 |
| 2 months | | | | | |
| WT scr.-shRNA vs. CMT1A scr.-shRNA | 28,19 | 17,62 to 38,75 | Yes | **** | <0,0001 |
| WT scr.-shRNA vs. CMT1A shRNA49 | 11,85 | 1,622 to 22,08 | Yes | * | 0,0256 |
| WT scr.-shRNA vs. CMT1A shRNA50 | 14,30 | 3,763 to 24,84 | Yes | ** | 0,0093 |
| CMT1A scr.-shRNA vs. CMT1A shRNA49 | -16,34 | -22,35 to -10,32 | Yes | **** | <0,0001 |
| CMT1A scr.-shRNA vs. CMT1A shRNA50 | -13,88 | -21,02 to -6,751 | Yes | *** | 0,0004 |
| CMT1A shRNA49 vs. CMT1A shRNA50 | 2,453 | -3,479 to 8,386 | No | ns | 0,5912 |
| 3 months | | | | | |
| WT scr.-shRNA vs. CMT1A scr.-shRNA | 32,34 | 20,29 to 44,38 | Yes | *** | 0,0001 |
| WT scr.-shRNA vs. CMT1A shRNA49 | 9,355 | -3,443 to 22,15 | No | ns | 0,1818 |
| WT scr.-shRNA vs. CMT1A shRNA50 | 13,38 | 0,3849 to 26,38 | Yes | * | 0,0431 |
| CMT1A scr.-shRNA vs. CMT1A shRNA49 | -22,98 | -31,66 to -14,31 | Yes | **** | <0,0001 |
| CMT1A scr.-shRNA vs. CMT1A shRNA50 | -18,95 | -28,10 to -9,811 | Yes | *** | 0,0005 |
| CMT1A shRNA49 vs. CMT1A shRNA50 | 4,028 | -6,474 to 14,53 | No | ns | 0,6736 |
| 6 months | | | | | |
| WT scr.-shRNA vs. CMT1A scr.-shRNA | 27,37 | 12,51 to 42,23 | Yes | *** | 0,0007 |

| | | | | | |
|---|---|---|---|---|---|
| WT scr.-shRNA vs. CMT1A shRNA49 | 2,244 | -14,20 to 18,69 | No | ns | 0,9764 |
| WT scr.-shRNA vs. CMT1A shRNA50 | 0,6541 | -12,28 to 13,59 | No | ns | 0,9984 |
| CMT1A scr.-shRNA vs. CMT1A shRNA49 | -25,12 | -40,89 to -9,365 | Yes | ** | 0,0025 |
| CMT1A scr.-shRNA vs. CMT1A shRNA50 | -26,72 | -38,36 to -15,07 | Yes | *** | 0,0003 |
| CMT1A shRNA49 vs. CMT1A shRNA50 | -1,590 | -15,75 to 12,57 | No | ns | 0,9826 |
| 9 months | | | | | |
| WT scr.-shRNA vs. CMT1A scr.-shRNA | 37,74 | 29,36 to 46,11 | Yes | **** | <0,0001 |
| WT scr.-shRNA vs. CMT1A shRNA49 | 7,325 | -1,824 to 16,47 | No | ns | 0,1336 |
| WT scr.-shRNA vs. CMT1A shRNA50 | 8,461 | -1,136 to 18,06 | No | ns | 0,0899 |
| CMT1A scr.-shRNA vs. CMT1A shRNA49 | -30,41 | -39,86 to -20,97 | Yes | **** | <0,0001 |
| CMT1A scr.-shRNA vs. CMT1A shRNA50 | -29,27 | -39,14 to -19,41 | Yes | **** | <0,0001 |
| CMT1A shRNA49 vs. CMT1A shRNA50 | 1,136 | -9,290 to 11,56 | No | ns | 0,9877 |
| 12 months | | | | | |
| WT scr.-shRNA vs. CMT1A scr.-shRNA | 36,01 | 24,62 to 47,41 | Yes | **** | <0,0001 |
| WT scr.-shRNA vs. CMT1A shRNA49 | 11,11 | -1,065 to 23,29 | No | ns | 0,0778 |
| WT scr.-shRNA vs. CMT1A shRNA50 | 11,48 | -4,036 to 26,99 | No | ns | 0,1761 |
| CMT1A scr.-shRNA vs. CMT1A shRNA49 | -24,90 | -35,27 to -14,53 | Yes | **** | <0,0001 |
| CMT1A scr.-shRNA vs. CMT1A shRNA50 | -24,53 | -39,12 to -9,942 | Yes | ** | 0,0024 |
| CMT1A shRNA49 vs. CMT1A shRNA50 | 0,3673 | -14,67 to 15,40 | No | ns | 0,9998 |

**Rotarod, Latency to fall (s)**

| | |
|---|---|
| Within each row, compare columns (simple effects within rows) | |
| Number of families | 6 |
| Number of comparisons per family | 6 |
| Alpha | 0,05 |

| Tukey's multiple comparisons test, two-sided | Mean Diff, | 95,00% CI of diff, | Significant? | Summary | Adjusted *p*-Value |
|---|---|---|---|---|---|

| | | | | | |
|---|---|---|---|---|---|
| 1 month | | | | | |
| WT scr.-shRNA vs. CMT1A scr.-shRNA | 28,14 | 9,336 to 46,95 | Yes | ** | 0,0044 |
| WT scr.-shRNA vs. CMT1A shRNA49 | 16,10 | -1,569 to 33,76 | No | ns | 0,0779 |
| WT scr.-shRNA vs. CMT1A shRNA50 | 25,57 | 8,638 to 42,50 | Yes | ** | 0,0038 |
| CMT1A scr.-shRNA vs. CMT1A shRNA49 | -12,05 | -32,66 to 8,561 | No | ns | 0,3481 |
| CMT1A scr.-shRNA vs. CMT1A shRNA50 | -2,571 | -22,67 to 17,53 | No | ns | 0,9803 |
| CMT1A shRNA49 vs. CMT1A shRNA50 | 9,476 | -9,653 to 28,61 | No | ns | 0,4828 |
| 2 months | | | | | |
| WT scr.-shRNA vs. CMT1A scr.-shRNA | 55,33 | 27,23 to 83,43 | Yes | *** | 0,0008 |
| WT scr.-shRNA vs. CMT1A shRNA49 | 23,81 | -3,970 to 51,59 | No | ns | 0,0949 |
| WT scr.-shRNA vs. CMT1A shRNA50 | 21,81 | -5,967 to 49,59 | No | ns | 0,1308 |
| CMT1A scr.-shRNA vs. CMT1A shRNA49 | -31,52 | -46,66 to -16,39 | Yes | *** | 0,0003 |
| CMT1A scr.-shRNA vs. CMT1A shRNA50 | -33,52 | -48,64 to -18,41 | Yes | *** | 0,0002 |
| CMT1A shRNA49 vs. CMT1A shRNA50 | -2,000 | -15,23 to 11,23 | No | ns | 0,9686 |
| 3 months | | | | | |
| WT scr.-shRNA vs. CMT1A scr.-shRNA | 79,45 | 31,53 to 127,4 | Yes | ** | 0,0034 |
| WT scr.-shRNA vs. CMT1A shRNA49 | 44,17 | -4,010 to 92,34 | No | ns | 0,0730 |
| WT scr.-shRNA vs. CMT1A shRNA50 | 35,21 | -13,19 to 83,62 | No | ns | 0,1736 |
| CMT1A scr.-shRNA vs. CMT1A shRNA49 | -35,29 | -58,81 to -11,76 | Yes | ** | 0,0039 |
| CMT1A scr.-shRNA vs. CMT1A shRNA50 | -44,24 | -69,13 to -19,35 | Yes | ** | 0,0011 |
| CMT1A shRNA49 vs. CMT1A shRNA50 | -8,952 | -35,37 to 17,46 | No | ns | 0,7483 |
| 6 months | | | | | |
| WT scr.-shRNA vs. CMT1A scr.-shRNA | 63,10 | 22,58 to 103,6 | Yes | ** | 0,0044 |
| WT scr.-shRNA vs. CMT1A shRNA49 | 25,86 | -14,21 to 65,92 | No | ns | 0,2245 |
| WT scr.-shRNA vs. CMT1A shRNA50 | 24,19 | -17,49 to 65,87 | No | ns | 0,3375 |
| CMT1A scr.-shRNA vs. CMT1A shRNA49 | -37,24 | -56,15 to -18,33 | Yes | *** | 0,0009 |
| CMT1A scr.-shRNA vs. CMT1A shRNA50 | -38,90 | -65,61 to -12,19 | Yes | ** | 0,0050 |
| CMT1A shRNA49 vs. CMT1A shRNA50 | -1,667 | -26,05 to 22,71 | No | ns | 0,9958 |
| 9 months | | | | | |
| WT scr.-shRNA vs. CMT1A scr.-shRNA | 70,10 | 38,41 to 101,8 | Yes | *** | 0,0004 |
| WT scr.-shRNA vs. CMT1A shRNA49 | 22,71 | -11,86 to 57,29 | No | ns | 0,2560 |
| WT scr.-shRNA vs. CMT1A shRNA50 | 25,90 | -5,965 to 57,77 | No | ns | 0,1200 |

| | | | | | |
|---|---|---|---|---|---|
| CMT1A scr.-shRNA vs. CMT1A shRNA49 | -47,38 | -73,21 to -21,55 | Yes | ** | 0,0011 |
| CMT1A scr.-shRNA vs. CMT1A shRNA50 | -44,19 | -63,51 to -24,87 | Yes | **** | <0,0001 |
| CMT1A shRNA49 vs. CMT1A shRNA50 | 3,190 | -22,96 to 29,35 | No | ns | 0,9817 |
| 12 months | | | | | |
| WT scr.-shRNA vs. CMT1A scr.-shRNA | 49,57 | 41,03 to 58,11 | Yes | **** | <0,0001 |
| WT scr.-shRNA vs. CMT1A shRNA49 | 13,33 | 4,051 to 22,62 | Yes | ** | 0,0053 |
| WT scr.-shRNA vs. CMT1A shRNA50 | 8,000 | -2,062 to 18,06 | No | ns | 0,1384 |
| CMT1A scr.-shRNA vs. CMT1A shRNA49 | -36,24 | -44,17 to -28,31 | Yes | **** | <0,0001 |
| CMT1A scr.-shRNA vs. CMT1A shRNA50 | -41,57 | -50,55 to -32,59 | Yes | **** | <0,0001 |
| CMT1A shRNA49 vs. CMT1A shRNA50 | -5,333 | -14,99 to 4,320 | No | ns | 0,3919 |

**Griptest, Strength (N)**

| | |
|---|---|
| Within each row, compare columns (simple effects within rows) | |
| Number of families | 6 |
| Number of comparisons per family | 6 |
| Alpha | 0,05 |

| Tukey's multiple comparisons test, two-sided | Mean Diff, | 95,00% CI of diff, | Significant? | Summary | Adjusted *p*-Value |
|---|---|---|---|---|---|
| 1 month | | | | | |
| WT scr.-shRNA vs. CMT1A scr.-shRNA | 0,7286 | -0,3728 to 1,830 | No | ns | 0,2503 |
| WT scr.-shRNA vs. CMT1A shRNA49 | 1,081 | 0,1810 to 1,981 | Yes | * | 0,0177 |
| WT scr.-shRNA vs. CMT1A shRNA50 | 1,071 | 0,1624 to 1,980 | Yes | * | 0,0199 |
| CMT1A scr.-shRNA vs. CMT1A shRNA49 | 0,3524 | -0,7189 to 1,424 | No | ns | 0,7558 |
| CMT1A scr.-shRNA vs. CMT1A shRNA50 | 0,3429 | -0,7346 to 1,420 | No | ns | 0,7742 |
| CMT1A shRNA49 vs. CMT1A shRNA50 | -0,009524 | -0,8709 to 0,8519 | No | ns | >0,9999 |

| | | | | | |
|---|---|---|---|---|---|
| 2 months | | | | | |
| WT scr.-shRNA vs. CMT1A scr.-shRNA | 3,900 | 2,475 to 5,325 | Yes | **** | <0,0001 |
| WT scr.-shRNA vs. CMT1A shRNA49 | 1,343 | 0,3521 to 2,334 | Yes | ** | 0,0086 |
| WT scr.-shRNA vs. CMT1A shRNA50 | 1,195 | 0,1976 to 2,193 | Yes | * | 0,0185 |
| CMT1A scr.-shRNA vs. CMT1A shRNA49 | -2,557 | -3,910 to -1,204 | Yes | ** | 0,0012 |
| CMT1A scr.-shRNA vs. CMT1A shRNA50 | -2,705 | -4,061 to -1,349 | Yes | *** | 0,0008 |
| CMT1A shRNA49 vs. CMT1A shRNA50 | -0,1476 | -0,9460 to 0,6507 | No | ns | 0,9450 |
| 3 months | | | | | |
| WT scr.-shRNA vs. CMT1A scr.-shRNA | 3,019 | 2,006 to 4,032 | Yes | **** | <0,0001 |
| WT scr.-shRNA vs. CMT1A shRNA49 | 1,367 | 0,2981 to 2,435 | Yes | * | 0,0120 |
| WT scr.-shRNA vs. CMT1A shRNA50 | 1,267 | 0,2339 to 2,299 | Yes | * | 0,0156 |
| CMT1A scr.-shRNA vs. CMT1A shRNA49 | -1,652 | -2,778 to -0,5263 | Yes | ** | 0,0045 |
| CMT1A scr.-shRNA vs. CMT1A shRNA50 | -1,752 | -2,847 to -0,6580 | Yes | ** | 0,0023 |
| CMT1A shRNA49 vs. CMT1A shRNA50 | -0,1000 | -1,242 to 1,042 | No | ns | 0,9935 |
| 6 months | | | | | |
| WT scr.-shRNA vs. CMT1A scr.-shRNA | 3,229 | 1,432 to 5,025 | Yes | ** | 0,0015 |
| WT scr.-shRNA vs. CMT1A shRNA49 | 0,7905 | -0,9637 to 2,545 | No | ns | 0,4874 |
| WT scr.-shRNA vs. CMT1A shRNA50 | 0,9857 | -0,8984 to 2,870 | No | ns | 0,4288 |
| CMT1A scr.-shRNA vs. CMT1A shRNA49 | -2,438 | -3,401 to -1,475 | Yes | *** | 0,0001 |
| CMT1A scr.-shRNA vs. CMT1A shRNA50 | -2,243 | -3,605 to -0,8809 | Yes | ** | 0,0020 |
| CMT1A shRNA49 vs. CMT1A shRNA50 | 0,1952 | -1,055 to 1,446 | No | ns | 0,9559 |
| 9 months | | | | | |
| WT scr.-shRNA vs. CMT1A scr.-shRNA | 2,881 | 1,834 to 3,928 | Yes | **** | <0,0001 |
| WT scr.-shRNA vs. CMT1A shRNA49 | 0,7857 | -0,09970 to 1,671 | No | ns | 0,0877 |
| WT scr.-shRNA vs. CMT1A shRNA50 | 0,1952 | -0,6762 to 1,067 | No | ns | 0,9050 |
| CMT1A scr.-shRNA vs. CMT1A shRNA49 | -2,095 | -3,066 to -1,125 | Yes | *** | 0,0002 |
| CMT1A scr.-shRNA vs. CMT1A shRNA50 | -2,686 | -3,645 to -1,727 | Yes | **** | <0,0001 |
| CMT1A shRNA49 vs. CMT1A shRNA50 | -0,5905 | -1,338 to 0,1566 | No | ns | 0,1416 |
| 12 months | | | | | |
| WT scr.-shRNA vs. CMT1A scr.-shRNA | 3,086 | 1,739 to 4,433 | Yes | *** | 0,0001 |
| WT scr.-shRNA vs. CMT1A shRNA49 | 0,9238 | -0,4650 to 2,313 | No | ns | 0,2496 |
| WT scr.-shRNA vs. CMT1A shRNA50 | 1,467 | 0,1451 to 2,788 | Yes | * | 0,0286 |

| | | | | | |
|---|---|---|---|---|---|
| CMT1A scr.-shRNA vs. CMT1A shRNA49 | -2,162 | -3,433 to -0,8903 | Yes | ** | 0,0014 |
| CMT1A scr.-shRNA vs. CMT1A shRNA50 | -1,619 | -2,807 to -0,4307 | Yes | ** | 0,0077 |
| CMT1A shRNA49 vs. CMT1A shRNA50 | 0,5429 | -0,6999 to 1,786 | No | ns | 0,5802 |

**Supplementary Table S3 List of the shRNAs and primers used and their sequences**

| Name | Genbank reference | Sequence (5'-3') |
|---|---|---|
| ShRNA sh1 | [NM_008885.2] | CGCGGTGCTAGTGTTGCTCTT |
| ShRNA sh1 | [NM_008885.2] | CACTGACTACTCCTATGGCTT |
| ShRNA shA | [NM_000304.4] | CCTGTTCTTCTGCCAACTCTT |
| ShRNA shB | [NM_000304.4] | GGCAATGGACACGCAACTGAT |
| Genotyping primers | [NM_008885.2] | Forward: GACAAACCCCAGACAGTTG<br>Reverse: CCAGAAAGCCAGGGAACTC |
| ITR-2 primers | | Forward: GGAACCCCTAGTGATGGAGTT<br>Reverse: CGGCCTCAGTGAGCGA<br>Taqman Probe used for vector titer:<br>FAM- CACTCCCTCTCTGCGCGCTCG-BBQ |
| Rat-specific *Pmp22* | [NM_17037.1] | Forward: GACAAACCCCAGATGGCC<br>Reverse: CCGCAGCCACCAGCTATTGGT |
| Mouse-specific *Pmp22* | [NM_008885.2] | Forward: GACAAACCCCAGACAGTTGA<br>Reverse: CAGGAGCCACCAGCTATTACT |
| Total *Pmp22* (mouse and rat) | | Forward: TGTACCACATCCGCCTTGG<br>Reverse: GAGCTGGCAGAAGAACAGGAAC |
| Rat *Rsp9* | [NM_031108.2] | Forward: ATCCGCCAACGTCACATC<br>Reverse: CCGCCACCATAAGGAGAAC |
| Rat *Actb* | [NM_031144.3] | Forward: CACCATGTACCCAGGCATT<br>Reverse: ACTTGCGCTCAGGAGGAG |
| Rat *Mpz* | [NM_017027.1] | Forward: TGTTGCTGCTGTTGCTCTTC<br>Reverse: TTGTGAAATTTCCCCTTCTCC |
| *GFP* | | Forward: ACTACAACAGCCACAACGTCTATATCA<br>Reverse: GGCGGATCTTGAAGTTCACC<br>Taqman Probe used for Biodistribution study:<br>FAM-CCGACAAGCAGAAGAACGGCATCA-TAMRA |
| Rat *Hprt1* | [NM_012583.2] | Forward: GCGAAAGTGGAAAAGCCAAGT<br>Reverse: GCCACATCAACAGGACTCTTGTAG<br>Taqman Probe used for biodistribution study:<br>JOE-CAAAGCCTAAAAGACAGCGGCAAGTTGAAT- TAMRA |
| Rat *Gstt2* | [NM_012796.2] | ForwarGCTCTACCTGGACCTGCTGT<br>Reverse: GAATTGCTCGCTCAAGTGCT |
| Rat *Ctsa* | [NM_001011959.2] | Forward: TTACAGAGCACGGTCCCTTC<br>Reverse: ATATACAGCATGTTGGCAATCAG |
| Rat *Nrg1-I* | [NM_001271125.2] | Forward: GGGAAGGGCAAGAAGAAGG<br>Reverse: TTTCGCACCGGAGCACTAGC |
| Rat *Pparg* | [NM_013124.3] | Forward: GGTGAAACTCTGGGAGATCCT<br>Reverse: AATGGCATCTCTGTGTCAAC |
| Rat *Fn3krp* | [NM_001107077.2] | Forward: CATTCAGCCCCAGATGGA<br>Reverse: CACGGAACAGGTCAGGAATC |
| Rat *Cda* | [NM_001108688.1] | Forward: AGCCTGCCGACAAGTCAT<br>Reverse: AACCTTCAGTGAATCTTCTGCAA |
| Rat *Enpp1* | [NM_053535.1] | Forward: TACCCCAAGTCATCCCAAAG<br>Reverse: AAGTCCATGATCGGCACAAT |
| Rat *Gria1* | [NM_031608.1] | Forward: AGGGATCGACATCCAGAGAG<br>Reverse: TGCACATTTCCTGTCAAACC |
| Rat *Anpep* | [NM_031012.1] | Forward: GCCCACCTGGAATCTGAA<br>Reverse: CTGGCGTGTTGACCTCGT |
