# Supplementary material for: AAV2/9-mediated silencing of PMP22 prevents the development of pathological features in a rat model of Charcot-Marie-Tooth disease 1 A
Source: Nat Commun. 2021 Apr 21;12:2356. doi: 10.1038/s41467-021-22593-3 (PMC8060274; doi:10.1038/s41467-021-22593-3)
Supplement: Supplementary file 3 — Description of Additional Supplementary Files [file 41467_2021_22593_MOESM3_ESM.docx]

**Description of Additional Supplementary Files**

**Title: Supplementary Movie 1**

**Description:** Illustration of the performance of a CMT1A ctr.sh rat on a narrow beam twelve months after injection

**Title: Supplementary Movie 2**

**Description:** Illustration of the performance of a CMT1A sh1 rat on a narrow beam twelve months after injection
