# Supplementary material for: AAV2/9-mediated silencing of PMP22 prevents the development of pathological features in a rat model of Charcot-Marie-Tooth disease 1 A
Source: Nat Commun. 2021 Apr 21;12:2356. doi: 10.1038/s41467-021-22593-3 (PMC8060274; doi:10.1038/s41467-021-22593-3)
Supplement: Supplementary file 6 — Reporting Summary [file 41467_2021_22593_MOESM6_ESM.pdf]

nature research

Corresponding author(s): Benoit Gautier, Nicolas Tricaud

Last updated by author(s): Mar 5, 2021

Reporting Summary

Nature Research wishes to improve the reproducibility of the work that we publish. This form provides structure for consistency and transparency in reporting. For further information on Nature Research policies, see our Editorial Policies and the Editorial Policy Checklist.

Statistics

For all statistical analyses, confirm that the following items are present in the figure legend, table legend, main text, or Methods section.

n/a	Confirmed	
☐	☒	The exact sample size (*n*) for each experimental group/condition, given as a discrete number and unit of measurement
☐	☒	A statement on whether measurements were taken from distinct samples or whether the same sample was measured repeatedly
☐	☒	The statistical test(s) used AND whether they are one- or two-sided<br>*Only common tests should be described solely by name; describe more complex techniques in the Methods section.*
☒	☐	A description of all covariates tested
☐	☒	A description of any assumptions or corrections, such as tests of normality and adjustment for multiple comparisons
☐	☒	A full description of the statistical parameters including central tendency (e.g. means) or other basic estimates (e.g. regression coefficient) AND variation (e.g. standard deviation) or associated estimates of uncertainty (e.g. confidence intervals)
☐	☒	For null hypothesis testing, the test statistic (e.g. *F*, *t*, *r*) with confidence intervals, effect sizes, degrees of freedom and *P* value noted<br>*Give P values as exact values whenever suitable.*
☒	☐	For Bayesian analysis, information on the choice of priors and Markov chain Monte Carlo settings
☒	☐	For hierarchical and complex designs, identification of the appropriate level for tests and full reporting of outcomes
☐	☒	Estimates of effect sizes (e.g. Cohen's *d*, Pearson's *r*), indicating how they were calculated

Our web collection on statistics for biologists contains articles on many of the points above.

Software and code

Policy information about availability of computer code

Data collection	Fluorescent imaging was acquired using Zen black and Zen Blue Editions (Zeiss)<br>Western blot images were acquired using Image Studio Lite (Li-Cor)<br>Electrophysiological data were acquired using LabChart (AD Instrument)<br>RT-qPCR data for the Biomarker studies were obtained using the software linked to the LightCycler 480 Systems (Roche Applied Science)<br>qPCR data for the biodistribution study were otained using the software linked to the StepOne Plus apparatus (Applied Biosytem, Thermo Fisher Scientific)
Data analysis	Fluorescent Images were processed using Zen Blue Edition (Zeiss) and Fiji (ImageJ)<br>Western blot analysis was performed using Image Studio Lite (Li-Cor)<br>Data were analysed with excel (Microsoft Office 2016) and GraphPad Prism 7<br>Statistical analysis and graphs were generated with GraphPad Prism 7<br>In the biomarker studies, R-package ade4 was used for PCA and correlation matrix and RStudio Version 1.0.153 was used for data plotting and statistical analysis

For manuscripts utilizing custom algorithms or software that are central to the research but not yet described in published literature, software must be made available to editors and reviewers. We strongly encourage code deposition in a community repository (e.g. GitHub). See the Nature Research guidelines for submitting code & software for further information.

Data

Policy information about availability of data

All manuscripts must include a data availability statement. This statement should provide the following information, where applicable:

- Accession codes, unique identifiers, or web links for publicly available datasets
- A list of figures that have associated raw data
- A description of any restrictions on data availability

All data and materials are available upon request. The source data underlying Tables 1, 2 and 3, Figs 2, 3, 4c,e-g, 5a–d and 6, and Supplementary Figs S6a-c, S7, S9 and Supplementary Table S1 are provided as a Source Data file. All exact p-values are available in the Source data file.
For Figure 1, Supplementary Figures S1, S2, S3, S4, S5, only illustrative images are presented with no data quantification

Field-specific reporting

Please select the one below that is the best fit for your research. If you are not sure, read the appropriate sections before making your selection.

☒ Life sciences ☐ Behavioural & social sciences ☐ Ecological, evolutionary & environmental sciences

For a reference copy of the document with all sections, see nature.com/documents/nr-reporting-summary-flat.pdf

Life sciences study design

All studies must disclose on these points even when the disclosure is negative.

Sample size	CMT1A rat experimental groups were sized according to the literature on this model to allow for statistical analysis: Fledrich, R. et al. Targeting myelin lipid metabolism as a potential therapeutic strategy in a model of CMT1A neuropathy. Nat Commun 9, 3025 (2018). Hajjar, H. et al. Label-free non-linear microscopy to measure myelin outcome in a rodent model of Charcot-Marie-Tooth diseases. Journal of Biophotonics 11, e201800186. (2018).Fledrich, R. et al. Soluble neuregulin-1 modulates disease pathogenesis in rodent models of Charcot-Marie-Tooth disease 1A. Nature Medicine 20, 1055–1061 (2014).Sereda, M. et al. A transgenic rat model of Charcot-Marie-Tooth disease. Neuron 16, 1049–1060 (1996). Mouse experimental groups were sized according to publications on the intranerve injection protocol that we used: Gonzalez, S., Fernando, R. N., Perrin-Tricaud, C. & Tricaud, N. In vivo introduction of transgenes into mouse sciatic nerve cells in situ using viral vectors. Nature Protocols 9, 1160–1169 (2014).<br>Animal number was minimized according to ethical guidelines
Data exclusions	Behavioral data originating from animals that died or had physical disabilities unrelated to CMT1A disease during the study were not used. No outliers were excluded from the study.
Replication	For in-vivo experiments, the numbers of animals in each cohort represents the replicat<br>In vitro experiments, such as transfection experiments, were replicated 3 times.
Randomization	Randomization was applied for in vivo experiments and behavioral tests. Rats were randomly assigned to the different experimental groups after genotyping. For the gene therapy assay, wild-type littermate (WT) and CMT1A male and female rats were randomly divided into four groups (WT ctr.sh, CMT1A ctr.sh, CMT1A sh1, CMT1A sh2) of sixteen rats each .Just before the sacrifice and the tissue collection, each rat received a specific number for all the experiments carried out afterwards. Rat samples from each group were then randomly allocated to each type of experiments (biochemical or histological).<br>For the in vivo experiments on mice the distribution was also random.<br>For the in vitro transfection studies, cells were randomly seeded into 6-well plates and randomly transfected by plasmids.
Blinding	Scientists who performed the animal experiments and analysis were blinded to the group's identity. The sacrifice/tissue collection step and the sample processing step (biochemical and histological studies) were performed by distincts scientists. Thus, the blinding was maintained until the end of the analysis except for the loading of the samples during Western blots in order to have a relevant disposition, for experiments in wich only a part of the rats was analysed (AAV neutralizing factor and AAV biodistribution); in these last cases, the allocation of the group was known to select the correct samples.

Reporting for specific materials, systems and methods

We require information from authors about some types of materials, experimental systems and methods used in many studies. Here, indicate whether each material, system or method listed is relevant to your study. If you are not sure if a list item applies to your research, read the appropriate section before selecting a response.

Materials & experimental systems

n/a	Involved in the study	
	x	Antibodies
	x	Eukaryotic cell lines
x		Palaeontology and archaeology
	x	Animals and other organisms
x		Human research participants
x		Clinical data
x		Dual use research of concern

Methods

n/a	Involved in the study	
x		ChIP-seq
x		Flow cytometry
x		MRI-based neuroimaging

Antibodies

Antibodies used

Antibodies used for for both immunohistochemistry on frozen sections and on teased fibers:
Mouse anti-Myelin Basic Protein (MBP-SMI-99, Millipore, reference NE1019, 1/1000),
Rat anti-Myelin Basic Protein (BIO-RAD, reference MCA409S, 1/1000)
Rabbit anti- β-Tubulin III (Tuj1, Sigma, reference T2200, 1/1000)
Rabbit anti-PMP22 (Sigma-Aldrich, SAB4502217, 1/500)
Rabbit anti-glial fibrillary acidic protein (GFAP, Dako, reference Z0334, 1/1000)
Rabbit anti-Neurofilament 200 (NF, Sigma, reference N4142, 1/500)
Mouse anti-E-cadherin (E-cad, BD Biosciences, reference 610182, 1/500)
Mouse anti-Sodium Channel (pan-Nav, Sigma, reference S8809, 1/500)
Goat anti-Contactin-1 (CNTN-1, RD System, reference AF904, 1/2000)
Donkey anti-mouse Alexa Fluor 594 (Thermofisher, reference A-21203, 1/1000)
Donkey anti-rabbit Alexa Fluor 594 (Thermofisher, reference A-21207, 1/1000)
Donkey anti-rat Alexa Fluor 594 (Thermofisher, reference A-21209, 1/1000)
Donkey anti-rabbit Alexa Fluor 647 (Thermofisher, reference A-31573, 1/1000)
Donkey anti-goat Alexa Fluor 647 (Thermofisher, reference A-21447, 1/1000)

Antibodies used for Western blot:
Rabbit anti-PMP22 (Sigma-Aldrich, SAB4502217, 1/750)
Mouse anti-β-Actin Clone AC-15 (Sigma-Aldrich, A1978, 1/10 000)
Goat anti MPZ (Thermo Fisher Scientific, PA5-18773, 1/1000)
Rabbit or mouse M2 anti Flag (Sigma-Aldrich, F7425, F1804, 1/1000).
IRDye 800CW donkey anti-rabbit (LI-COR Biosciences, 925-32213, 1/15000)
IRDye 680RD donkey anti-mouse (LI-COR Biosciences, 925-68072, 1/15000)
IRDye 800CW donkey anti-goat (LI-COR Biosciences, 925-32214, 1/15000).

Validation

All primary antibodies were commercially available and validated by the manufacturer:

Mouse anti-Myelin Basic Protein (MBP-SMI-99, Millipore, reference NE1019): validation for IHC in Mammals tissue (https://www.merckmillipore.com/FR/fr/product/Anti-Myelin-Basic-Protein-Mouse-mAb-SMI-99,EMD_BIO-NE1019)

Rat anti-Myelin Basic Protein (BIO-RAD, reference MCA409S): validation for IHC in Mammals tissue (https://www.bio-rad-antibodies.com/monoclonal/cow-bovine-mbp-antibody-12-mca409.html?f=s%2Fn)

Rabbit anti- β-Tubulin III (Tuj1, Sigma, reference T2200): validation for IHC in mouse and rat tissue (https://www.sigmaaldrich.com/catalog/product/sigma/t2200?lang=fr&region=FR)

Rabbit anti-PMP22 (Sigma-Aldrich, SAB4502217): validation for Western blot and IHC in mouse, rat and human cells and tissues (https://www.sigmaaldrich.com/catalog/product/sigma/sab4502217?lang=fr&region=FR)

Rabbit anti-glial fibrillary acidic protein (GFAP, Dako, reference Z0334) validation for IHC (https://www.agilent.com/en/product/immunohistochemistry/antibodies-controls/primary-antibodies/glial-fibrillary-acidic-protein-(concentrate)-76683#specifications)

Rabbit anti-Neurofilament 200 (NF, Sigma, reference N4142): validation IHC in mouse, rat (https://www.sigmaaldrich.com/catalog/product/sigma/n4142?lang=fr&region=FR)

Mouse anti-E-cadherin (E-cad, BD Biosciences, reference 610182): validation in IHC in mouse and rat (https://www.bdbiosciences.com/eu/applications/research/stem-cell-research/cancer-research/human/purified-mouse-anti-e-cadherin-36e-cadherin/p/610182)

Mouse anti-Sodium Channel (pan-Nav, Sigma, reference S8809): validation for IHC in Mammals (https://www.sigmaaldrich.com/catalog/product/sigma/s8809?lang=fr&region=FR)

Goat anti-Contactin-1 (CNTN-1, RD System, reference AF904): validation for IHC in mouse and rat (https://www.rndsystems.com/

products/human-mouse-rat-contactin-1-antibody_af904)

Mouse anti-β-Actin Clone AC-15 (Sigma-Aldrich, A1978): validation for Western blot in mouse, rat and human cells (https://www.sigmaaldrich.com/catalog/product/sigma/a1978?lang=fr&region=FR)

Goat anti MPZ (Thermo Fisher Scientific, PA5-18773): validation for Western blot in mouse , rat and human cells and tissues (https://www.thermofisher.com/antibody/product/MPZ-Antibody-Polyclonal/PA5-18773)

Rabbit or mouse M2 anti Flag (Sigma-Aldrich, F7425, F1804): validation for Western blot in many cell lines after transfection (https://www.sigmaaldrich.com/catalog/search?term=F7425&interface=All&N=0&mode=match%20partialmax&lang=fr&region=FR&focus=product; https://www.sigmaaldrich.com/catalog/search?term=f7425&interface=All&N=0&mode=match%20partialmax&lang=fr&region=FR&focus=papers; https://www.sigmaaldrich.com/catalog/search?term=F1804&interface=All&N=0&mode=match%20partialmax&lang=fr&region=FR&focus=product)

Eukaryotic cell lines

Policy information about cell lines

Cell line source(s)	HEK293 cell line from ATCC<br>RT4-D6P2T cell line from ATCC<br>MSC80 from Boutry, J. M. et al. Establishment and characterization of a mouse Schwann cell line which produces myelin in vivo. J Neurosci Res 32, 15–26 (1992). Provided by Dr A. VanEvercooren.
Authentication	None of the cell lines was authenticated
Mycoplasma contamination	All cell lines were tested negative for mycoplasma
Commonly misidentified lines<br>(See ICLAC register)	Non applicable

Animals and other organisms

Policy information about studies involving animals; ARRIVE guidelines recommended for reporting animal research

Laboratory animals

C57BL/6 female mice were purchased from Janvier Labs (France). These mice were used to evaluate the transduction pattern of AAV2/9 and AAV2/rh10 after intra-nerve injection. A cohort of three adult mice (2-3 months old) and three pups (P2-P3) were injected with each AAV vectors as described in the section "vector delivery".

A CMT1A rat breeding colony was established from a gift of CMT1A rats from Max Planck Institute of Experimental Medicine, Goettingen, Germany. Littermates and CMT1A rats, both gender, were identified using PCR on DNA isolated from the tail.
For the transduction pattern, a cohort of three adult rats (both gender, 2-3 months old) and three pups (both gender,P6-P7) were injected with each AAV vectors as described in the section "vector delivery".

For the gene therapy assay, wild-type littermate (WT) and CMT1A male and female rats were randomly divided into four groups (WT ctr.sh, CMT1A ctr.sh, CMT1A sh1, CMT1A sh2) of sixteen rats each. At three months of age half of the rats in each group (eight per group) were sacrificed for biochemical and biodistribution studies. All the others were kept until twelve months of age to study the efficiency of the gene therapy on the sensory-motor behavior and NCV at different time points post injection (1, 2, 3, 6, 9 and 12 months). Finally, these animals were sacrificed for histological and biochemical studies.

Rodents were maintained on a 12 h dark, 12 h ligh cycle with an ambient temperature of 21-22 °C and an ambient humidity between 40 and 60 %.

Two juvenile cynomolgus macaques (Macaca fascicularis, two females, 3.7 years old/ 4.3 kg and 2.3 years old/2.9 kg) were included in the study. Animals were part of the the MIRCen colony (CEA Fontenay-aux-roses, France). Progenitors were imported from a licensed primate breeding centers on Mauritius and Philippines. The experiments were performed in an authorized user facility (Ministère de l'Agriculture, number 92–032-02). Non-human primates remained under veterinary care during the full study. Animals were tested

	negative for anti-AAV2/9 or anti-AAV2/rh10 antibodies before the treatment.
Wild animals	No wild animal was used
Field-collected samples	No field collection
Ethics oversight	All animal experiments were approved by the local ethic committee and the ministère de la recherche et de l'enseignement supérieur (authorization 2017032115087316 and 2016091313354892 for rodents and 2015061911295753v2 for NHP). All the procedures were performed in accordance with the French regulation for the animal procedure (French decree 2013-118) and with specific European Union guidelines for the protection of animal welfare (Directive 2010/63/EU). on-human primate experiments were performed in an authorized user facility (Ministère de l'Agriculture, number 92–032-02). Animals remained under veterinary care during the full study.

Note that full information on the approval of the study protocol must also be provided in the manuscript.
